# Supplementary material for: Determining sex differences in aortic valve myofibroblast responses to drug combinations identified using a digital medicine platform
Source: Sci Adv. 2025 Jun 6;11(23):eadu2695. doi: 10.1126/sciadv.adu2695 (PMC12143354; doi:10.1126/sciadv.adu2695)
Supplement: Supplementary file 1 — Figs. S1 to S16 Tables S1 to S16 IDentif.AI Code Legend for data S1 References [file sciadv.adu2695_sm.pdf]

Science Advances
AAAS

Supplementary Materials for

Determining sex differences in aortic valve myofibroblast responses to drug combinations identified using a digital medicine platform

Brandon J. Vogt *et al.*

Corresponding author: Brian A. Aguado, baguado@ucsd.edu; Dean Ho, biedh@nus.edu.sg; Edward Kai-Hua Chow, edwardkchow@nus.edu.sg

Sci. Adv. **11**, eadu2695 (2025)
DOI: 10.1126/sciadv.adu2695

The PDF file includes:

Figs. S1 to S16
Tables S1 to S16
IDentif.AI Code
Legend for data S1
References

Other Supplementary Material for this manuscript includes the following:

Data S1

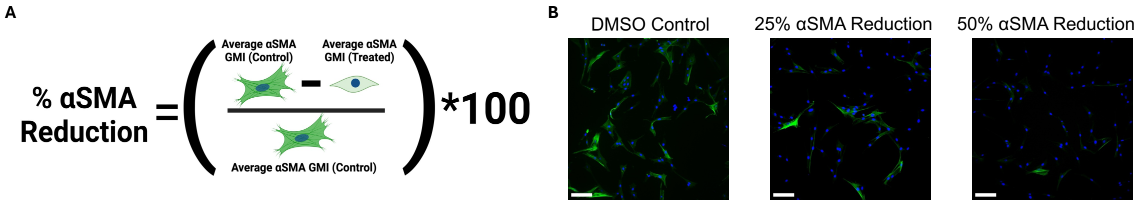

Figure S1. Overview of percent αSMA reduction output. (**A**) Schematic defining how percent αSMA reduction is calculated. GMI=Gradient mean intensity. (**B**) Representative immunofluorescent images of male VICs cultured on hydrogels with αSMA stained in green and DAPI stained in blue. Scale bar = 100 μm.

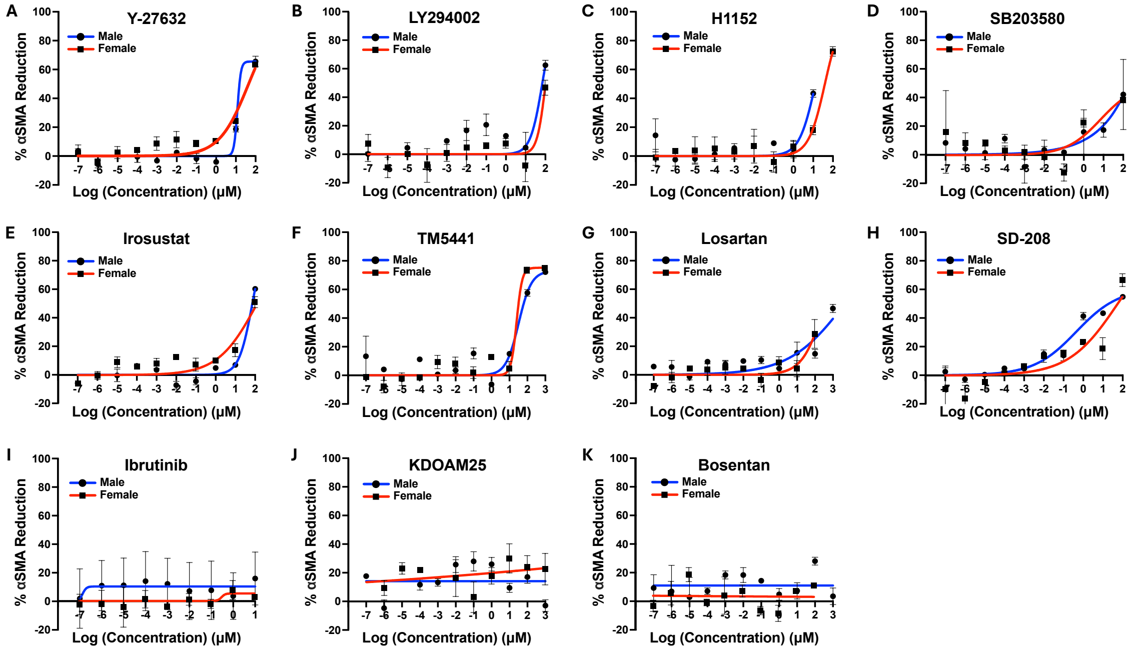

Figure S2. Eight out of eleven anti-fibrotic drugs inhibit myofibroblast activation in male and female VICs on TCPS. (**A-K**) Percent αSMA reduction in male and female VICs cultured on TCPS at doses ranging from 10^{-7} μM up to 10^{3} μM for (**A**) Y-27632, (**B**) LY294002, (**C**) H1152, (**D**) SB203580, (**E**) Irosustat, (**F**) TM5441, (**G**) Losartan, (**H**) SD-208, (**I**) Ibrutinib, (**J**) KDOAM25, and (**K**) Bosentan (n = 2 wells). Each data point is plotted as mean ± standard error of the mean. The best fit line shown was generated using a nonlinear regression curve fit with GraphPad Prism.

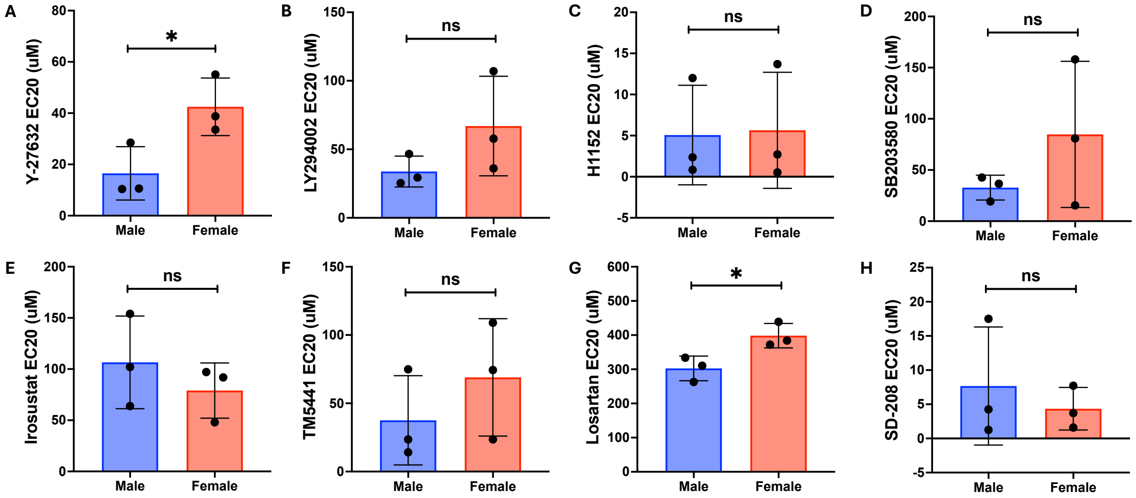

Figure S3. Male VICs cultured on TCPS are more responsive to Y-27632 and Losartan relative to female VICs. (**A-H**) Male versus female EC_{20} values for VICs cultured on TCPS with (**A**) Y-27632, (**B**) LY294002, (**C**) H1152, (**D**) SB203580, (**E**) Irosustat, (**F**) TM5441, (**G**) Losartan, and (**H**) SD-208 (N = 3 biological replicates). Data is plotted as mean ± standard deviation. Statistical significance was determined by an unpaired two-tailed t-test with Welch's correction and indicated as *=P<0.05. Y-27632 and Losartan showed a significant difference in efficacy between male and female VICs.

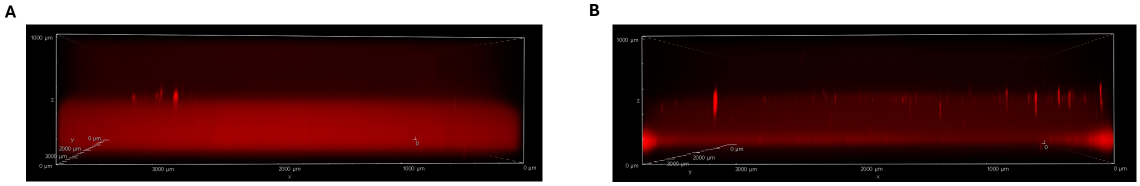

Figure S4. Soft and stiff hydrogels formed in a 96-well plate are flat. (**A-B**) Representative immunofluorescent side-view images of (**A**) soft and (**B**) stiff hydrogels stained with Cy5.

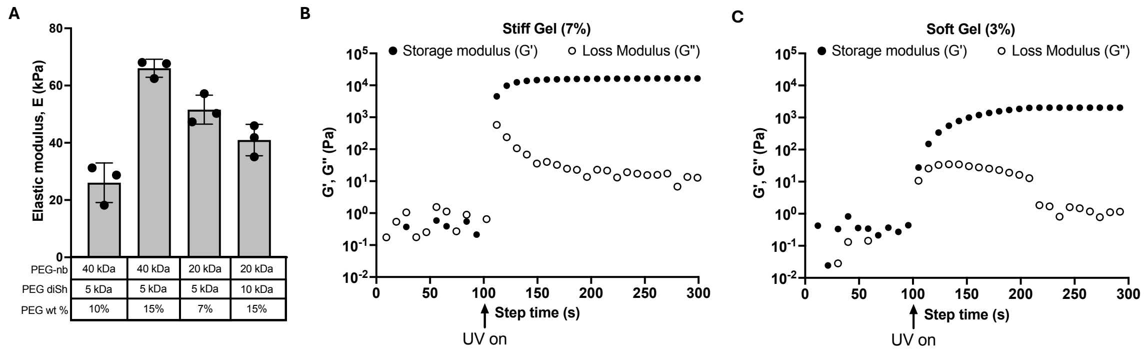

Figure S5. Rheological measurements of various hydrogel compositions. (**A**) Rheology of candidate stiff gels comprised of different PEG monomers. Data shown as mean ± standard deviation (n = 3 gel samples). (**B-C**) Storage and loss moduli of optimized (**B**) stiff and (**C**) soft hydrogel precursor solution formed with 20 kDa PEG-norbornene (PEG-nb) and 5 kDa PEG dithiol (PEG-diSh) crosslinker (n = 1; representative replicate shown).

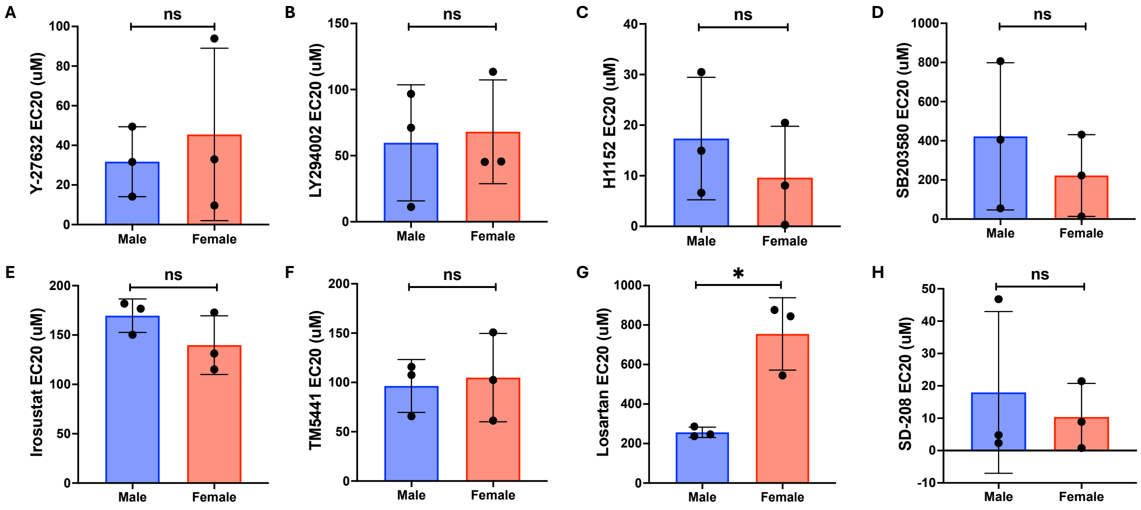

Figure S6. Male VICs cultured on stiff hydrogels are more responsive to Losartan relative to female VICs. (**A-H**) Male versus female EC_{20} values for VICs cultured on stiff hydrogels with (**A**) Y-27632, (**B**) LY294002, (**C**) H1152, (**D**) SB203580, (**E**) Irosustat, (**F**) TM5441, (**G**) Losartan, and (**H**) SD-208 (N = 3 biological replicates). Data is plotted as mean ± standard deviation. Statistical significance was determined by an unpaired two-tailed t-test with Welch's correction and indicated as *=$P<0.05$. Losartan showed a significant difference in efficacy between male and female VICs.

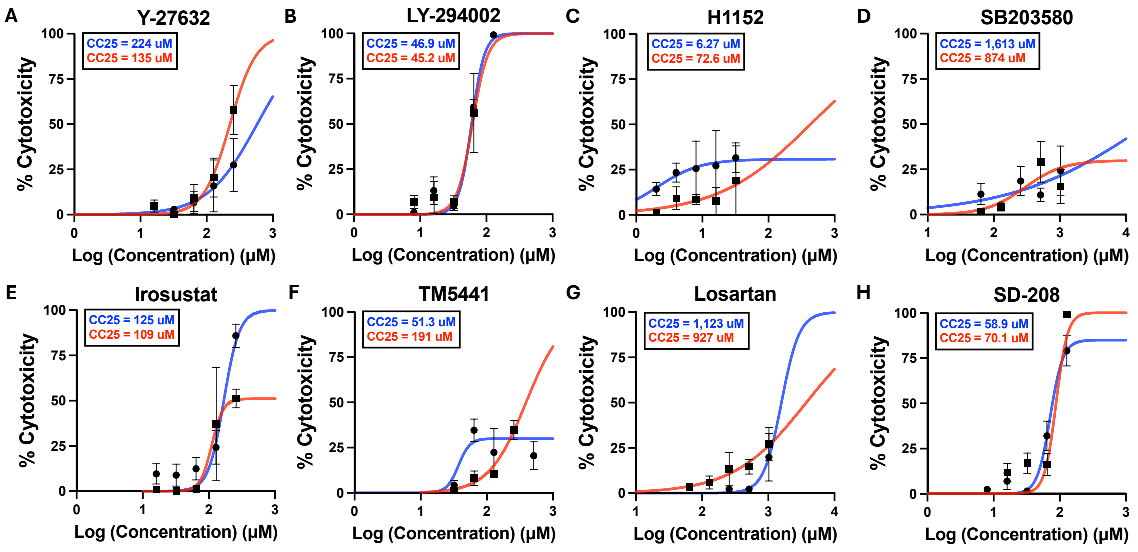

Figure S7. Characterizing cytotoxicity of inhibitors in male and female VICs cultured on stiff hydrogels. (**A-H**) Percent cytotoxicity in male VICs (blue) and female VICs (red) cultured on stiff gels for (**A**) Y-27632, (**B**) LY294002, (**C**) H1152, (**D**) SB203580, (**E**) Irosustat, (**F**) TM5441, (**G**) Losartan, and (**H**) SD-208 (n = 3 gels). Data is plotted as mean ± standard error of the mean. The best fit line shown was generated using a nonlinear regression curve fit with GraphPad Prism.

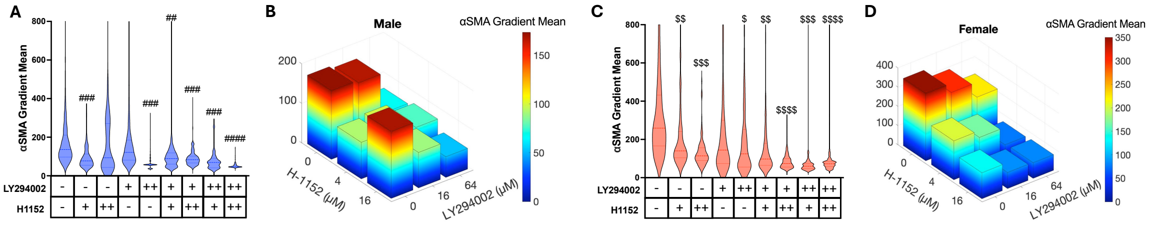

Figure S8. The 96-well hydrogel platform captures the combinatorial effects of H-1152 and LY294002 on male and female VICs. (**A,C**) Violin plot showing the distribution of (**A**) male or (**C**) female VIC single cell αSMA gradient mean values for combinations of a moderate or high dose of H-1152 and/or LY294002 on stiff hydrogels. A minimum of 300 cells were used per condition. (**B,D**) 3-D bar plot showing the mean αSMA gradient value for (**B**) male VICs or (**D**) female VICs at each experimental condition. - = no drug, + = moderate drug dose, ++ = high drug dose. Statistical significance was determined by one-way ANOVA with Tukey posttests ($P<0.0001$) and effect size using the Cohen's d-value indicated as ##=d>0.5, ###=d>0.8, ####=d>1.2 relative to male control and $=d>0.2, $$=d>0.5, $$$=d>0.8, $$$$=d>1.2 relative to female control.

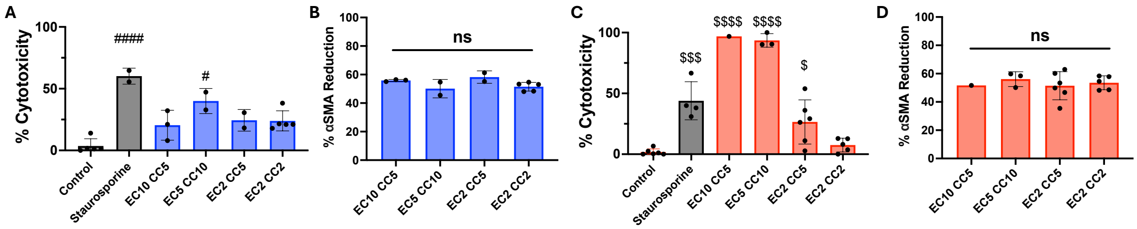

Figure S9. A combination of all eight inhibitors at EC_2/CC_2/10% C_{max} doses are non-cytotoxic while still maintaining maximum therapeutic efficacy in male and female VICs cultured on stiff hydrogels. (**A,C**) Bar graph showing percent cytotoxicity of (**A**) male or (**C**) female VICs with different doses of eight drug combinations on stiff hydrogels. (**B,D**) Bar graph showing the percent αSMA reduction for (**B**) male VICs or (**D**) female VICs with different doses of eight drug combinations on stiff hydrogels (n = 1-6 gels). All drug combinations were additionally limited by 10% C_{max}. Data is plotted as mean ± standard deviation. Statistical significance was determined by one-way ANOVA with Tukey posttests. For male VICs, #=P<0.05 and ####=P<0.0001 relative to control. For female VICs, $=P<0.05, $$$=P<0.001, and $$$$=P<0.0001 relative to control.

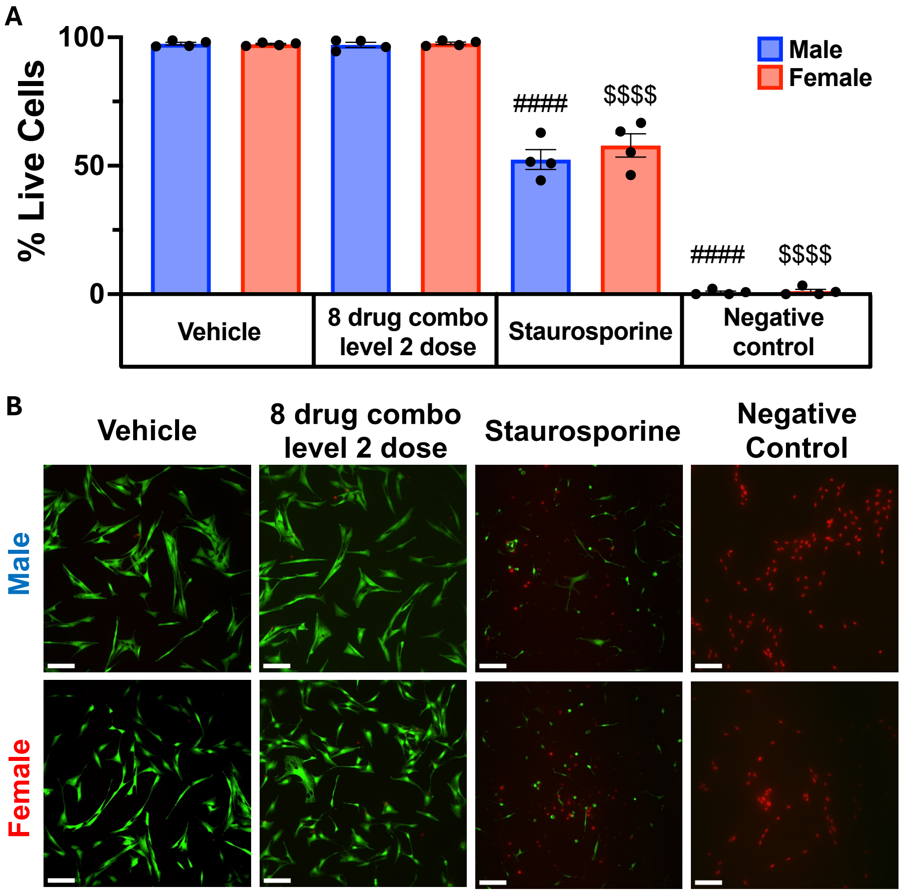

Figure S10. Using a LIVE/DEAD cell imaging kit confirms that using all eight inhibitors together at the L2 dose is non-cytotoxic in male and female VICs cultured on stiff hydrogels. (**A**) Bar graph showing the percentage of live male or female VICs exposed to the combination comprising of all eight drugs at L2 doses and controls (n = 4 gels) (**B**) Representative immunofluorescent images with live cells stained in green (Calcein) and dead cells stained in red (BOBO-3 Iodide). Scale bar = 100 µm. Data is plotted as mean ± standard deviation. Statistical significance was determined by one-way ANOVA with Tukey posttests. ####=P<0.0001 relative to male vehicle control and $$$$=P<0.0001 relative to female vehicle control. The negative control group was treated with chilled 70% ethanol for 15 minutes prior to imaging.

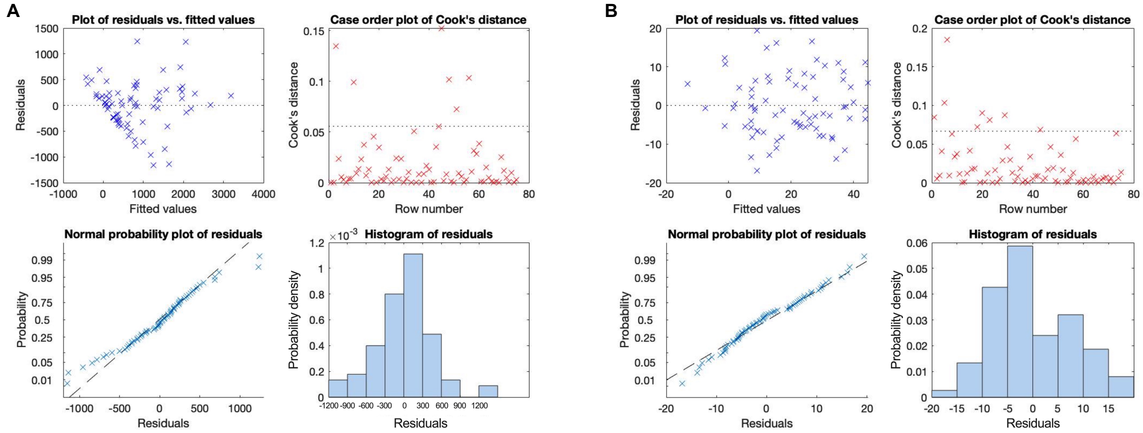

Figure S11. Residual-based outlier analysis for percent αSMA reduction data. (**A-B**) A series of outlier analysis for prospectively acquired percent αSMA reduction data resulting from the 59 OACD combinations for both (**A**) male and (**B**) female VICs (n = 2-4 gels). No outliers were detected, and all data were included in the IDentif.AI analysis.

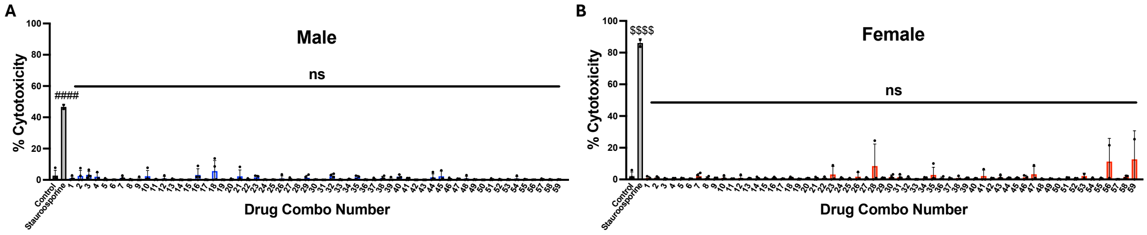

Figure S12. Drug combinations used for IDentif.AI analysis of male and female VICs on stiff hydrogels are non-cytotoxic. (**A-B**) Percent cytotoxicity of all 59 combinations used for IDentif.AI analysis of (**A**) male VICs or (**B**) female VICs on stiff hydrogels with positive and negative controls (n = 2-4 gels). Data is plotted as mean ± standard deviation. Statistical significance was determined by one-way ANOVA with Tukey posttests and indicated as ###=P<0.001 relative to male control and $$$$=P<0.0001 relative to female control.

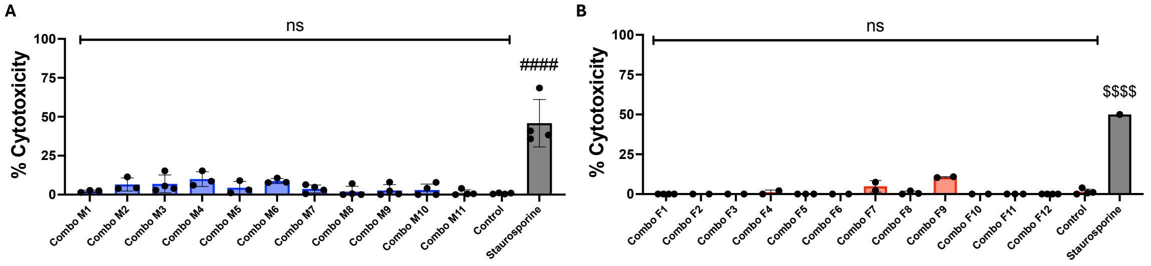

Figure S13. Drug combinations used for male and female IDentif.AI validations on stiff hydrogels are non-cytotoxic. (**A-B**) Percent cytotoxicity of all combinations tested to validate the IDentif.AI model for (**A**) male VICs or (**B**) female VICs on stiff hydrogels with positive and negative controls (n = 2-4 gels). Statistical significance was determined by one-way ANOVA with Tukey posttests and indicated as ####=P<0.001 relative to male control and $$$$=P<0.0001 relative to female control.

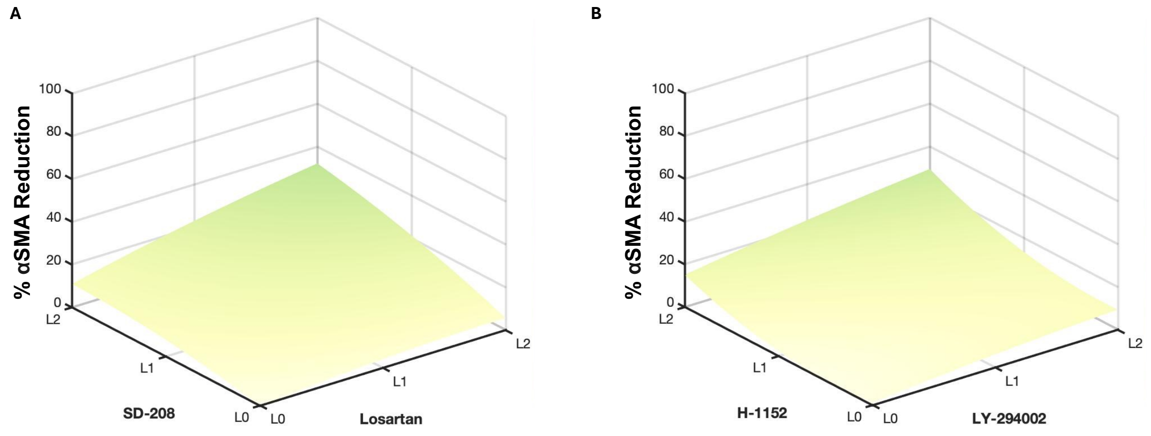

Figure S14. Interaction surfaces of IDentif.AI-detected interactions for male and female VIC analyses. (**A-B**) Interaction surfaces for (**A**) Losartan/SD-208 and (**B**) LY294002/H1152 combinations. The predicted interaction of Losartan/SD-208 in male VICs indicated that when both drugs achieve L2 concentrations, the combination may exhibit interactions to enhance efficacy. Similarly, for female VICs, LY294002/H1152 may interact to improve efficacy at L2 concentrations.

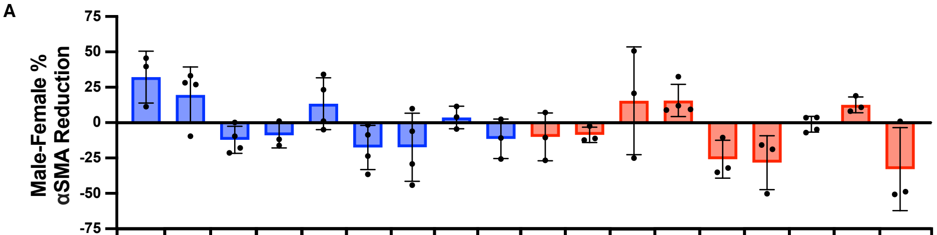

Drug Combo #	M1	M2	M3	M4	M5	M6	M7	M8	M9	F1	F2	F3	F4	F5	F6	F7	F8	F9
Significance	ns	ns	ns	ns	ns	ns	ns	ns	ns	ns	ns	ns	ns	ns	ns	ns	ns	ns
Male % αSMA Reduction	32 ±5%	12 ±8%	37 ±6%	17 ±13%	11 ±7%	37 ±7%	23 ±10%	22 ±5%	27 ±14%	6 ±20%	-6 ±9%	51 ±5%	41 ±4%	21 ±27%	13 ±21%	18 ±9%	33 ±15%	15 ±9%
Female % αSMA Reduction	0 ±15%	-8 ±25%	49 ±11%	28 ±18%	-2 ±17%	55 ±12%	40 ±15%	16 ±5%	39 ±22%	14 ±5%	3 ±7%	36 ±24%	25 ±14%	46 ±27%	41 ±2%	19 ±3%	20 ±16%	48 ±36%

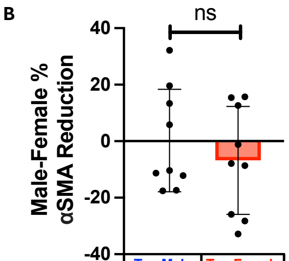

	Top Male Combos	Top Female Combos
Male % αSMA Reduction	24 ±10%	21 ±18%
Female % αSMA Reduction	24 ±23%	28 ±16%

Figure S15. Top male and female drug combinations are not sex-specific on stiff hydrogels. (**A**) Percent αSMA reduction difference (male-female) between male and female VICs for top male drug combinations (M1-M9) and top female drug combinations (F1-F9) on stiff hydrogels (n = 3-4 gels). (**B**) Percent αSMA reduction difference between male and female VICs cultured with top male and female combinations with nine drug combinations per group. Data is plotted as mean ± standard deviation. Statistical significance was determined for (**A**) by one-way by one sample t-test against a theoretical mean of zero to represent the vehicle control and for (**B**) by unpaired two-tailed t-test with Welch's correction.

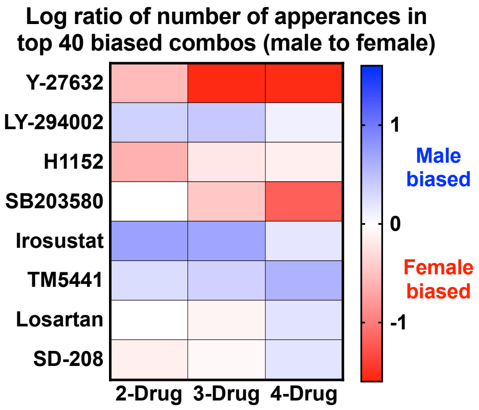

Figure S16. Compositions of the top 40 male-biased and female-biased combinations. Heat map showing the log ratio of the number of drug appearances in the top 40 male-biased combinations to the number of drug appearances in the top 40 female-biased combinations for 2-drug, 3-drug, and 4-drug combinations.

Table S1. Eleven inhibitors and corresponding therapeutic targets to reduce myofibroblast activation.

Drug	Therapeutic Target
Y-27632	Rho-kinase (ROCK) 1 and ROCK 2
LY294002	Phosphoinositide 3-kinase α (PI3Kα), PI3Kδ, PI3Kβ
H1152	ROCK2
SB203580	p38 mitogen-activated protein kinase
Irosustat	Steroid sulfatase
TM5441	Plasminogen activator inhibitor 1
Losartan	Angiotensin II type 1 receptor
SD-208	Transforming growth factor beta receptor type 1
Ibrutinib	Bruton’s tyrosine kinase and BMX non-receptor tyrosine kinase
KDOAM25	Lysine demethylase 5A (KDM5A), KDM5B, KDM5C, KDM5D
Bosentan	Endothelin 1

Table S2. Level 1 (L1) and level 2 (L2) drug dosing for male VICs cultured on stiff hydrogels. The L1 concentration was selected based on the lowest of: EC_1, CC_1, and 5% of C_{max}. Similarly, the L2 concentration was selected based on the lowest of EC_2, CC_2, and 10% of C_{max}. The EC and CC values were derived from dose response curves in Figs. 3 and S7. These selection criteria ensure that the selected L1/L2 doses are clinically relevant and non-toxic. The C_{max} values are derived from FDA documents and clinical studies. Note that some selected drugs are investigational drugs that do not have available C_{max}.

**Drug**	**EC1 (μM)**	**5% Cmax (μM)**	**CC1 (μM)**	**Level 1 Dose**	**EC2 (μM)**	**10% Cmax (μM)**	**CC2 (μM)**	**Level 2 Dose**	**Reference**
Y-27632	0.558	-	10.8	**0.558**	1.34	-	19.9	**1.34**	
LY294002	12.8	-	21.5	**12.8**	17.6	-	25.2	**17.6**	
H1152	1.96	-	0.162	**0.162**	2.82	-	0.28	**0.28**	
SB203580	24.8	-	0.413	**0.413**	40.8	-	2.18	**2.18**	
Irosustat	92.6	0.0106	48.5	**0.0106**	106.3	0.0212	58.7	**0.0212**	(79)
TM5441	4.96	-	18.9	**4.96**	9.3	-	21.9	**9.3**	
Losartan	18.7	0.0265	378	**0.0265**	32.9	0.053	470	**0.053**	(80)
SD-208	0.0582	-	26.6	**0.0582**	0.188	-	31.1	**0.188**	

Table S3. Level 1 (L1) and level 2 (L2) drug dosing for female VICs cultured on stiff hydrogels. The L1 concentration was selected based on the lowest of: EC_1, CC_1, and 5% of C_{max}. Similarly, the L2 concentration was selected based on the lowest of EC_2, CC_2, and 10% of C_{max}. The EC and CC values were derived from dose response curves in Figs. 3 and S7. These selection criteria ensure that the selected L1/L2 doses are clinically relevant and non-toxic. The C_{max} values are derived from FDA documents and clinical studies. Note that some selected drugs are investigational drugs that do not have available C_{max}.

Drug	EC1 (µM)	5% Cmax (µM)	CC1 (µM)	Level 1 Dose	EC2 (µM)	10% Cmax (µM)	CC2 (µM)	Level 2 Dose	Reference
Y-27632	1.31	-	27.5	**1.31**	2.81	-	37.9	**2.81**	
LY294002	8.63	-	17.7	**8.63**	13.4	-	21.3	**13.4**	
H1152	0.0765	-	0.26	**0.0765**	0.198	-	0.806	**0.198**	
SB203580	7.56	-	29.8	**7.56**	14.5	-	48.9	**14.5**	
Irosustat	74.4	0.0106	50.2	**0.0106**	84.7	0.0212	57.9	**0.0212**	(79)
TM5441	24.8	-	19.7	**19.7**	33.6	-	31.1	**31.1**	
Losartan	154	0.0265	11.1	**0.0265**	219	0.053	27	**0.053**	(80)
SD-208	0.0061	-	34.8	**0.0061**	0.0275	-	40.1	**0.0275**	

Table S4. 8-drug resolution IV orthogonal array composite design (OACD). The -1, 0, and 1 in the OACD represent the level 0, level 1, and level 2 concentrations, respectively.

Combination	Y-27632	LY294002	H1152	SB203580	Irosustat	TM5441	Losartan	SD-208
1	-1	-1	-1	-1	-1	1	-1	-1
2	1	-1	-1	-1	-1	-1	1	1
3	-1	1	-1	-1	-1	-1	1	-1
4	1	1	-1	-1	-1	1	-1	1
5	-1	-1	1	-1	-1	-1	-1	1
6	1	-1	1	-1	-1	1	1	-1
7	-1	1	1	-1	-1	1	1	1
8	1	1	1	-1	-1	-1	-1	-1
9	-1	-1	-1	1	-1	-1	-1	-1
10	1	-1	-1	1	-1	1	1	1
11	-1	1	-1	1	-1	1	1	-1
12	1	1	-1	1	-1	-1	-1	1
13	-1	-1	1	1	-1	1	-1	1
14	1	-1	1	1	-1	-1	1	-1
15	-1	1	1	1	-1	-1	1	1
16	1	1	1	1	-1	1	-1	-1
17	-1	-1	-1	-1	1	1	1	1
18	1	-1	-1	-1	1	-1	-1	-1
19	-1	1	-1	-1	1	-1	-1	1
20	1	1	-1	-1	1	1	1	-1
21	-1	-1	1	-1	1	-1	1	-1
22	1	-1	1	-1	1	1	-1	1
23	-1	1	1	-1	1	1	-1	-1
24	1	1	1	-1	1	-1	1	1
25	-1	-1	-1	1	1	-1	1	1
26	1	-1	-1	1	1	1	-1	-1
27	-1	1	-1	1	1	1	-1	1
28	1	1	-1	1	1	-1	1	-1
29	-1	-1	1	1	1	1	1	-1
30	1	-1	1	1	1	-1	-1	1
31	-1	1	1	1	1	-1	-1	-1
32	1	1	1	1	1	1	1	1
33	-1	-1	-1	-1	-1	-1	-1	-1
34	-1	0	0	-1	-1	0	1	1
35	-1	1	1	-1	-1	1	0	0
36	-1	-1	0	1	0	-1	0	0
37	-1	0	1	1	0	0	-1	-1
38	-1	1	-1	1	0	1	1	1
39	-1	-1	1	0	1	-1	1	1
40	-1	0	-1	0	1	0	0	0
41	-1	1	0	0	1	1	-1	-1
42	0	-1	0	0	-1	0	-1	0
43	0	0	1	0	-1	1	1	-1
44	0	1	-1	0	-1	-1	0	1
45	0	-1	1	-1	0	0	0	1
46	0	0	-1	-1	0	1	-1	0
47	0	1	0	-1	0	-1	1	-1
48	0	-1	-1	1	1	0	1	-1
49	0	0	0	1	1	1	0	1
50	0	1	1	1	1	-1	-1	0
51	1	-1	1	1	-1	1	-1	1
52	1	0	-1	1	-1	-1	1	0
53	1	1	0	1	-1	0	0	-1
54	1	-1	-1	0	0	1	0	-1
55	1	0	0	0	0	-1	-1	1
56	1	1	1	0	0	0	1	0
57	1	-1	0	-1	1	1	1	0
58	1	0	1	-1	1	-1	0	-1
59	1	1	-1	-1	1	0	-1	1

Table S5. Percent αSMA reduction for all 59 combinations used to generate the IDentif.AI model fit for male VICs cultured on hydrogels. n= 3-4 gels.

Combination	Replicate 1	Replicate 2	Replicate 3	Replicate 4	Average	Standard Deviation	Standard Error
1	-10.73	16.81	36.20	30.17	18.11	20.86	10.43
2	46.82	27.64	33.34	16.42	31.06	12.64	6.32
3	31.59	42.82	34.98	53.51	40.72	9.74	4.87
4	64.89	36.33	28.66	-	43.29	19.09	11.02
5	-1.51	15.24	-8.39	2.32	1.91	9.93	4.96
6	0.97	17.38	17.78	-	12.04	9.59	5.54
7	59.68	57.16	32.89	37.34	46.77	13.61	6.81
8	6.05	14.17	12.02	2.83	8.77	5.24	2.62
9	2.77	17.21	28.83	29.22	19.51	12.47	6.24
10	47.60	53.29	48.97	36.33	46.55	7.23	3.62
11	-1.57	-2.70	-0.50	-	-1.59	1.10	0.63
12	-12.34	27.59	39.04	28.04	20.58	22.58	11.29
13	39.66	16.08	17.49	47.14	30.09	15.67	7.84
14	13.21	19.58	31.93	9.09	18.45	9.97	4.98
15	-8.79	10.50	5.82	19.58	6.78	11.85	5.92
16	44.99	40.00	36.44	31.82	38.31	5.57	2.79
17	7.74	-6.64	-6.87	26.52	5.19	15.78	7.89
18	11.12	11.01	36.56	18.90	19.40	12.02	6.01
19	19.69	-5.01	10.56	-2.30	5.74	11.52	5.76
20	45.60	27.76	35.99	14.34	30.92	13.25	6.62
21	0.58	19.47	30.75	-	16.93	15.24	8.80
22	39.66	33.12	21.27	40.45	33.62	8.87	4.43
23	58.14	50.08	50.86	-	53.03	4.44	2.56
24	51.11	36.03	39.64	-	42.26	7.88	4.55
25	19.76	18.42	27.46	31.54	24.29	6.26	3.13
26	54.85	37.84	36.44	3.08	33.05	21.66	10.83
27	-1.58	19.53	-24.74	-2.81	-2.40	18.08	9.04
28	9.15	15.85	24.66	46.25	23.98	16.15	8.08
29	55.38	39.88	12.99	6.70	28.74	22.86	11.43
30	19.00	10.90	20.11	-	16.67	5.03	2.90
31	-1.88	13.17	10.37	-	7.22	8.00	4.62
32	53.52	50.14	41.22	35.50	45.10	8.24	4.12
33	24.72	14.98	5.30	-26.60	4.60	22.26	11.13
34	3.84	24.95	23.26	-	17.35	11.73	6.77
35	50.48	35.56	37.14	-	41.06	8.20	4.73
36	7.28	14.22	6.29	16.26	11.01	4.97	2.48
37	-0.42	29.96	31.95	29.32	22.70	15.45	7.73
38	50.77	32.53	54.93	4.48	35.68	22.96	11.48
39	7.51	4.54	-11.03	-	0.34	9.96	5.75
40	-4.50	35.68	-3.92	-	9.09	23.03	13.30
41	33.87	27.81	34.51	23.61	29.95	5.20	2.60
42	29.61	25.24	19.88	16.90	22.91	5.65	2.82
43	22.21	13.64	-4.62	3.20	8.61	11.75	5.88
44	42.68	42.13	48.86	32.76	41.61	6.64	3.32
45	55.72	53.68	47.57	-	52.32	4.24	2.45
46	58.73	37.46	25.79	33.94	38.98	14.04	7.02
47	36.00	30.84	42.89	58.53	42.06	12.04	6.02
48	20.30	21.25	5.09	-7.08	9.89	13.52	6.76
49	43.55	39.34	37.05	43.37	40.83	3.18	1.59
50	14.53	24.68	24.62	30.42	23.56	6.61	3.31
51	31.66	20.74	15.72	16.61	21.18	7.32	3.66
52	35.00	39.87	24.91	18.11	29.47	9.81	4.90
53	13.59	25.14	34.65	42.27	28.91	12.39	6.19
54	20.89	23.25	9.82	-11.44	10.63	15.83	7.92
55	26.85	-4.60	28.61	-7.82	10.76	19.65	9.83
56	6.20	6.90	0.92	-	4.67	3.27	1.89
57	17.20	15.57	-1.33	-3.10	7.08	10.78	5.39
58	28.84	11.18	-0.67	8.07	11.86	12.38	6.19
59	23.15	-3.37	-19.73	3.32	0.84	17.75	8.87

Table S6. Percent αSMA reduction for individual level 1 and level 2 drug doses used to generate the IDentif.AI model fit for male VICs cultured on stiff hydrogels. n = 3-4 gels. SD=Standard deviation. SE=Standard error.

	Drug	Replicate 1	Replicate 2	Replicate 3	Replicate 4	Average	SD	SE
Level 1 dose	Y-27632	12.82	-12.17	2.34	-2.04	0.24	10.36	5.18
	LY294002	-9.56	18.87	11.27	-	6.86	14.72	8.50
	H1152	-2.68	-10.05	-14.68	7.31	-5.03	9.59	4.80
	SB203580	18.18	8.93	-10.77	-	5.45	14.78	8.53
	Irosustat	6.97	1.96	-6.40	-	0.84	6.75	3.90
	TM5441	-3.67	25.74	-26.46	-	-1.46	26.17	15.11
	Losartan	1.05	10.96	7.66	2.66	5.58	4.56	2.28
	SD-208	31.08	-7.59	20.32	-	14.60	19.96	11.53
Level 2 dose	Y-27632	30.83	-6.93	-11.04	-3.38	2.37	19.23	9.62
	LY294002	7.61	0.98	-3.19	16.60	5.50	8.63	4.32
	H1152	23.58	3.59	-18.16	-6.37	0.66	17.68	8.84
	SB203580	9.92	-17.32	26.88	-4.78	3.67	19.06	9.53
	Irosustat	0.11	-17.93	13.54	6.97	0.67	13.56	6.78
	TM5441	13.93	0.51	6.21	-11.91	2.18	10.89	5.44
	Losartan	-2.70	1.72	5.92	-0.97	0.99	3.76	1.88
	SD-208	14.55	-15.09	-11.20	14.27	0.63	15.99	7.99

Table S7. Coefficients and model fit parameters used for IDentif.AI analysis for male VICs cultured on stiff hydrogels. All terms are shown after a square transformation to improve the overall model fit. Statistical significance determined by F-test. *=P<0.05, **=P<0.01, ***=P<0.001, ****=P<0.0001.

Term	Estimate	Statistical Significance
Intercept	1380.2	****
Y-27632	177.10	*
LY294002	250.12	***
H1152	132.26	ns
SB203580	-82.663	ns
Irosustat	26.218	ns
TM5441	355.91	****
Losartan	77.384	ns
SD-208	183.28	*
Y-27632:SB203580	172.55	*
LY294002:SB203580	-258.25	**
LY294002:TM5441	140.01	ns
H1152:SB203580	-156.00	*
H1152:Irosustat	276.25	***
H1152:TM5441	225.02	**
Irosustat:SD-208	-179.49	*
TM5441:Losartan	-212.45	**
Losartan:SD-208	253.83	**
$Y\text{-}27632^2$	-766.35	***
$SB203580^2$	634.05	**
$Irosustat^2$	-349.22	ns
Degrees of Freedom	54	
R^2	0.748	
Adjusted R^2	0.655	
F-statistic vs constant model	8.02	****

Table S8. Top IDentif.AI predicted effective 2-drug, 3-drug, and 4-drug combinations and two low ranked drug combinations for male VICs selected for *in vitro* validation. Drug doses are indicated as -1 for no drug, 0 for level 1 dose, and 1 for level 2 dose. Abbreviations: Y-27632 (Y27); LY294002 (LY); H1152 (H11); SB203580 (SB); Irosustat (Iro); TM5441 (TM); Losartan (Los); SD-208 (SD).

Combo #	Y27	LY	H11	SB	Iro	TM	Los	SD	Predicted % αSMA Reduction
M1	-1	1	-1	-1	-1	1	-1	-1	36.72
M2	0	1	-1	-1	-1	-1	-1	-1	34.31
M3	-1	-1	-1	-1	-1	-1	1	1	31.65
M4	0	1	-1	-1	-1	1	-1	-1	45.11
M5	-1	1	1	-1	-1	1	-1	-1	42.06
M6	-1	1	-1	-1	-1	-1	1	1	40.60
M7	-1	1	1	-1	1	1	-1	-1	51.37
M8	0	1	1	-1	-1	1	-1	-1	49.64
M9	0	1	-1	-1	-1	-1	1	1	48.38
M10	-1	1	-1	1	-1	-1	-1	-1	2.86
M11	1	-1	1	1	-1	-1	-1	-1	-2.23

Table S9. Percent αSMA reduction for all 59 combinations used to generate the IDentif.AI model fit for female VICs cultured on hydrogels. n = 2-4 gels.

Combination	Replicate 1	Replicate 2	Replicate 3	Replicate 4	Average	Standard Deviation	Standard Error
1	29.24	24.65	31.88	-	28.59	3.65	2.11
2	34.90	41.86	43.89	-	40.22	4.71	2.72
3	-5.17	21.00	22.53	38.61	19.24	18.12	9.06
4	51.11	49.67	41.40	40.55	45.68	5.48	2.74
5	24.36	35.36	28.29	20.31	27.08	6.41	3.20
6	36.61	47.31	44.28	44.87	43.27	4.63	2.31
7	-5.17	19.86	24.81	27.72	16.81	15.01	7.50
8	30.46	33.54	29.26	9.42	25.67	10.98	5.49
9	2.19	-7.11	24.36	24.81	11.06	16.07	8.04
10	44.60	41.80	35.87	15.64	34.48	13.08	6.54
11	-2.09	6.23	19.68	22.31	11.53	11.49	5.75
12	36.04	39.58	36.22	36.96	37.20	1.64	0.82
13	14.50	-15.20	21.79	-	7.03	19.59	11.31
14	-7.11	14.78	-0.44	5.15	3.10	9.26	4.63
15	37.24	23.67	-1.18	20.25	20.00	15.91	7.95
16	43.71	56.04	51.32	-	50.36	6.22	3.59
17	7.09	-18.11	-11.61	-	-7.54	13.08	7.55
18	18.09	34.11	51.14	22.53	31.47	14.75	7.37
19	11.99	15.81	-4.66	-	7.71	10.88	6.28
20	25.61	-11.72	-13.32	-	0.19	22.03	12.72
21	28.80	19.97	1.16	14.78	16.18	11.57	5.78
22	18.43	27.89	6.00	-	17.44	10.98	6.34
23	41.47	57.08	66.18	-	54.91	12.50	7.22
24	42.63	37.20	29.71	-	36.51	6.48	3.74
25	32.76	14.86	32.94	-	26.86	10.39	6.00
26	43.91	47.85	36.36	-	42.71	5.84	3.37
27	28.57	15.88	22.83	-	22.43	6.36	3.67
28	46.38	39.47	44.28	-	43.38	3.54	2.05
29	47.79	45.74	46.70	23.84	41.02	11.48	5.74
30	41.70	30.31	11.69	29.71	28.35	12.40	6.20
31	28.69	18.16	34.02	8.46	22.33	11.36	5.68
32	42.41	48.94	40.33	-	43.89	4.49	2.59
33	2.65	-5.55	5.28	-3.22	-0.21	5.03	2.52
34	20.31	6.72	19.77	13.49	15.07	6.37	3.19
35	35.52	15.58	9.95	37.97	24.76	14.07	7.03
36	3.25	5.70	0.61	-6.63	0.73	5.33	2.67
37	23.66	25.04	26.78	10.13	21.40	7.62	3.81
38	15.10	29.47	-5.19	-	13.13	17.42	10.06
39	-2.08	9.59	5.52	-	4.35	5.93	3.42
40	-12.32	-8.33	18.54	-	-0.70	16.79	9.69
41	16.24	29.41	11.33	-	18.99	9.35	5.40
42	24.38	27.80	28.33	23.78	26.07	2.32	1.16
43	26.24	19.11	12.17	25.16	20.67	6.48	3.24
44	30.13	45.02	30.91	-	35.35	8.38	4.84
45	15.40	7.37	31.28	-	18.02	12.17	7.02
46	29.92	21.96	45.56	-	32.48	12.00	6.93
47	34.06	41.16	45.10	-	40.11	5.60	3.23
48	28.20	22.25	18.10	17.15	21.42	5.03	2.52
49	36.18	30.52	31.38	21.01	29.77	6.35	3.17
50	38.03	38.05	46.46	-	40.85	4.86	2.81
51	-8.45	5.04	9.64	19.19	6.35	11.50	5.75
52	37.54	17.88	23.86	19.41	24.67	8.95	4.47
53	4.24	40.01	-	-	22.13	25.30	17.89
54	27.45	29.07	18.39	-	24.97	5.76	3.32
55	11.17	14.81	26.05	-	17.34	7.75	4.48
56	36.58	55.48	-	-	46.03	13.37	9.45
57	-17.06	0.08	-1.60	-	-6.19	9.45	5.46
58	23.42	20.58	40.44	-	28.14	10.74	6.20
59	37.53	21.09	-	-	29.31	11.63	8.22

Table S10. Percent αSMA reduction for individual level 1 and level 2 drug doses used to generate the IDentif.AI model fit for female VICs cultured on stiff hydrogels. n = 3-4 gels. SD=Standard deviation. SE=Standard error.

	Drug	Replicate 1	Replicate 2	Replicate 3	Replicate 4	Average	SD	SE
Level 1 dose	Y-27632	26.45	26.29	34.04		28.93	4.43	2.56
	LY294002	17.97	11.18	18.50	-9.65	9.50	13.19	6.60
	H1152	11.18	6.02	6.93	-	8.04	2.76	1.59
	SB203580	9.86	4.49	-7.28	-	2.35	8.77	5.06
	Irosustat	27.17	7.47	-5.77	-	9.62	16.58	9.57
	TM5441	2.03	-0.44	17.70	-13.20	1.52	12.68	6.34
	Losartan	24.32	-7.12	3.81	-	7.00	15.96	9.21
	SD-208	-7.55	18.40	1.71	-	4.19	13.15	7.59
Level 2 dose	Y-27632	22.31	22.17	21.88	-	22.12	0.22	0.13
	LY294002	-9.86	20.44	-20.52	20.93	2.75	21.17	10.58
	H1152	24.96	11.99	2.25	10.38	12.39	9.40	4.70
	SB203580	5.32	-22.94	-0.01	-8.57	-6.55	12.33	6.17
	Irosustat	-2.33	-6.74	7.41	-	-0.55	7.24	4.18
	TM5441	-4.05	-2.76	-16.11	-	-7.64	7.36	4.25
	Losartan	6.05	-1.18	7.70	-3.07	2.37	5.30	2.65
	SD-208	8.87	12.26	5.32	-	8.81	3.47	2.00

Table S11. Coefficients and model fit parameters used for IDentif.AI analysis for female VICs cultured on stiff hydrogels. Statistical significance determined by F-test. *=P<0.05, **=P<0.01, ***=P<0.001, ****=P<0.0001.

Term	Estimate	Statistical Significance
Intercept	23.784	****
Y-27632	7.493	****
LY294002	4.8144	***
H1152	2.6284	*
SB203580	1.4806	ns
Irosustat	1.4956	ns
TM5441	1.1921	ns
Losartan	-1.636	ns
SD-208	-0.50618	ns
Y-27632:H1152	-3.944	**
LY294002:H1152	2.7807	*
H1152:SB203580	-3.0194	*
H1152:Irosustat	2.8757	*
H1152:TM5441	2.7529	*
H1152:SD-208	-3.6649	**
SB203580:Irosustat	5.0124	***
SB203580:TM5441	2.7493	*
TM5441:Losartan	-3.5073	**
TM5441:SD-208	-2.7553	*
Y-27632^2	-7.5615	*
H-1152^2	6.7199	*
Degrees of Freedom	54	
R^2	0.745	
Adjusted R^2	0.651	
F-statistic vs constant model	7.89	****

Table S12. Top IDentif.AI predicted effective 2-drug, 3-drug, and 4-drug combinations and three low ranked drug combinations for female VICs selected for *in vitro* validation. Drug doses are indicated as -1 for no drug, 0 for level 1 dose, and 1 for level 2 dose. Abbreviations: Y-27632 (Y27); LY294002 (LY); H1152 (H11); SB203580 (SB); Irosustat (Iro); TM5441 (TM); Losartan (Los); SD-208 (SD).

Combo #	Y27	LY	H11	SB	Iro	TM	Los	SD	Predicted % αSMA Reduction
F1	1	-1	-1	-1	-1	-1	-1	1	39.96
F2	-1	1	1	-1	-1	-1	-1	-1	30.15
F3	-1	-1	1	-1	-1	1	-1	-1	29.87
F4	-1	1	1	-1	-1	1	-1	-1	45.06
F5	1	1	-1	-1	-1	-1	-1	1	44.03
F6	1	-1	-1	-1	-1	-1	1	1	43.70
F7	0	1	1	-1	-1	1	-1	-1	56.17
F8	1	1	-1	-1	-1	-1	1	1	47.77
F9	-1	1	1	-1	0	1	-1	-1	44.42
F10	-1	-1	-1	-1	1	0	-1	-1	-5.57
F11	-1	-1	1	1	-1	-1	-1	1	-6.48
F12	-1	-1	-1	-1	1	1	1	0	-13.50

Table S13. Top IDentif.AI predicted differentially effective 2-drug, 3-drug, and 4-drug combinations. Drug doses are indicated as -1 for no drug, 0 for level 1 dose, and 1 for level 2 dose. Abbreviations: Y-27632 (Y27); LY294002 (LY); H1152 (H11); SB203580 (SB); Irosustat (Iro); TM5441 (TM); Losartan (Los); SD-208 (SD).

Combo #	Y27	LY	H11	SB	Iro	TM	Los	SD	Predicted Male-Female % αSMA Reduction
MB1	-1	**1**	-1	-1	**0**	-1	-1	-1	24.52
MB2	-1	**1**	-1	-1	**1**	**1**	-1	-1	34.55
MB3	-1	**1**	-1	-1	-1	**1**	**1**	**1**	38.11
FB1	**1**	-1	-1	-1	-1	-1	-1	**1**	-28.64
FB2	**1**	-1	-1	**0**	-1	-1	-1	**0**	-30.47
FB3	**1**	-1	-1	**0**	**0**	-1	-1	**1**	-32.79

Table S14. Percent αSMA reduction for individual level 1 (L1) and level 2 (L2) drug doses used to generate the top male drug combinations (M1-M9) for male VICs cultured on soft hydrogels and the top female drug combinations (F1-F9) for female VICs cultured on soft hydrogels. n = 3 gels. SD=Standard deviation. SE=Standard error.

	Drug	Replicate 1	Replicate 2	Replicate 3	Average	SD	SE
Male VICs	Y-27632 L1	14.89	-1.01	-21.62	-2.58	18.31	10.57
	LY294002 L2	1.43	17.82	20.43	13.23	10.30	5.95
	H1152 L2	1.41	-12.02	-4.50	-5.04	6.73	3.89
	Irosustat L2	-22.30	0.19	-9.03	-10.38	11.31	6.53
	TM5441 L2	13.72	5.76	20.02	13.16	7.15	4.13
	Losartan L2	8.08	-6.63	0.99	0.81	7.35	4.25
	SD-208 L2	23.83	21.48	-28.58	5.58	29.61	17.09
Female VICs	Y-27632 L1	15.13	22.59	-28.07	3.22	27.35	15.79
	Irosustat L1	16.03	5.89	-7.52	4.80	11.81	6.82
	Y-27632 L2	-4.49	-2.04	31.24	8.24	19.96	11.52
	LY294002 L2	20.47	1.72	11.87	11.35	9.38	5.42
	H1152 L2	7.97	-6.43	8.59	3.37	8.50	4.91
	TM5441 L2	33.03	24.30	-5.86	17.16	20.40	11.78
	Losartan L2	14.68	16.94	-23.42	2.73	22.68	13.09
	SD-208 L2	14.92	4.43	8.76	9.37	5.27	3.04

Table S15. Percent αSMA reduction for individual level 1 (L1) and level 2 (L2) drug doses used to generate the top male drug combinations (M1-M9) for male VICs cultured on TCPS and the top female drug combinations (F1-F9) for female VICs cultured on TCPS. n = 3 wells. SD=Standard deviation. SE=Standard error.

	Drug	Replicate 1	Replicate 2	Replicate 3	Average	SD	SE
Male VICs	Y-27632 L1	6.92	9.51	3.02	6.48	3.27	1.89
	LY294002 L2	-7.63	-5.40	-1.71	-4.91	2.99	1.73
	H1152 L2	7.75	-3.14	-4.87	-0.09	6.84	3.95
	Irosustat L2	-6.73	-0.87	4.15	-1.15	5.45	3.15
	TM5441 L2	23.92	13.63	17.53	18.36	5.19	3.00
	Losartan L2	-0.96	-2.55	-0.47	-1.33	1.09	0.63
	SD-208 L2	7.58	6.50	9.32	7.80	1.42	0.82
Female VICs	Y-27632 L1	-0.09	2.84	-1.62	0.37	2.27	1.31
	Irosustat L1	6.87	-0.62	-0.59	1.88	4.32	2.49
	Y-27632 L2	-3.62	-1.82	-0.76	-2.07	1.45	0.84
	LY294002 L2	4.94	-9.22	-20.68	-8.32	12.83	7.41
	H1152 L2	4.07	-1.32	4.14	2.30	3.13	1.81
	TM5441 L2	6.54	8.20	10.37	8.37	1.92	1.11
	Losartan L2	2.37	2.77	-0.99	1.39	2.07	1.19
	SD-208 L2	4.11	6.07	-1.02	3.05	3.66	2.11

Table S16. Forward and reverse primers for RT-qPCR. All primers are shown from 5'-3' end.

Gene	Forward Primer (5'-3')	Reverse Primer (5'-3')
RPL30	AGATTTCCTCAAGGCTGGGC	GCTGGGGTACAAGCAGACTC
ACTA2	GCAAACAGGAATACGATGAAGCC	AACACATAGGTAACGAGTCAGAGC
COL1A1	GGGCAAGACAGTGATTGAATACA	GGATGGAGGGAGTTTACAGGAA

IDentif.AI Code

%Load OACD/Monotherapy (L1/L2) Data and Respective % αSMA reduction Data

data =
[

Y-27632	LY-294002	H-1152	SB203580	Irosustat	TM5441	Losartan	SD-208	Average
-1	-1	-1	-1	-1	1	-1	-1	28.6
1	-1	-1	-1	-1	-1	1	1	40.2
-1	1	-1	-1	-1	-1	1	-1	19.2
1	1	-1	-1	-1	1	-1	1	45.7
-1	-1	1	-1	-1	-1	-1	1	27.1
1	-1	1	-1	-1	1	1	-1	43.3
-1	1	1	-1	-1	1	1	1	16.8
1	1	1	-1	-1	-1	-1	-1	25.7
-1	-1	-1	1	-1	-1	-1	-1	11.1
1	-1	-1	1	-1	1	1	1	34.5
-1	1	-1	1	-1	1	1	-1	11.5
1	1	-1	1	-1	-1	-1	1	37.2
-1	-1	1	1	-1	1	-1	1	7.0
1	-1	1	1	-1	-1	1	-1	3.1
-1	1	1	1	-1	-1	1	1	20.0
1	1	1	1	-1	1	-1	-1	50.4
-1	-1	-1	-1	1	1	1	1	-7.5
1	-1	-1	-1	1	-1	-1	-1	31.5
-1	1	-1	-1	1	-1	-1	1	7.7
1	1	-1	-1	1	1	1	-1	0.2
-1	-1	1	-1	1	-1	1	-1	16.2
1	-1	1	-1	1	1	-1	1	17.4
-1	1	1	-1	1	1	-1	-1	54.9
1	1	1	-1	1	-1	1	1	36.5
-1	-1	-1	1	1	-1	1	1	26.9
1	-1	-1	1	1	1	-1	-1	42.7
-1	1	-1	1	1	1	-1	1	22.4
1	1	-1	1	1	-1	1	-1	43.4
-1	-1	1	1	1	1	1	-1	41.0
1	-1	1	1	1	-1	-1	1	28.4
-1	1	1	1	1	-1	-1	-1	22.3
1	1	1	1	1	1	1	1	43.9
-1	-1	-1	-1	-1	-1	-1	-1	-0.2
-1	0	0	-1	-1	0	1	1	15.1
-1	1	1	-1	-1	1	0	0	24.8
-1	-1	0	1	0	-1	0	0	0.7
-1	0	1	1	0	0	-1	-1	21.4
-1	1	-1	1	0	1	1	1	13.1
-1	-1	1	0	1	-1	1	1	4.3
-1	0	-1	0	1	0	0	0	-0.7
-1	1	0	0	1	1	-1	-1	19.0
0	-1	0	0	-1	0	-1	0	26.1
0	0	1	0	-1	1	1	-1	20.7
0	1	-1	0	-1	-1	0	1	35.4
0	-1	1	-1	0	0	0	1	18.0
0	0	-1	-1	0	1	-1	0	32.5
0	1	0	-1	0	-1	1	-1	40.1
0	-1	-1	1	1	0	1	-1	21.4
0	0	0	1	1	1	0	1	29.8
0	1	1	1	1	-1	-1	0	40.8
1	-1	1	1	-1	1	-1	1	6.4
1	0	-1	1	-1	-1	1	0	24.7
1	1	0	1	-1	0	0	-1	22.1
1	-1	-1	0	0	1	0	-1	25.0
1	0	0	0	0	-1	-1	1	17.3
1	1	1	0	0	0	1	0	46.0
1	-1	0	-1	1	1	1	0	-6.2
1	0	1	-1	1	-1	0	-1	28.1
1	1	-1	-1	1	0	-1	1	29.3
0	-1	-1	-1	-1	-1	-1	-1	28.9
-1	0	-1	-1	-1	-1	-1	-1	9.5
-1	-1	0	-1	-1	-1	-1	-1	8.0
-1	-1	-1	0	-1	-1	-1	-1	2.4
-1	-1	-1	-1	0	-1	-1	-1	9.6
-1	-1	-1	-1	-1	0	-1	-1	1.5
-1	-1	-1	-1	-1	-1	0	-1	7.0
-1	-1	-1	-1	-1	-1	-1	0	4.2
1	-1	-1	-1	-1	-1	-1	-1	22.1
-1	1	-1	-1	-1	-1	-1	-1	2.7
-1	-1	1	-1	-1	-1	-1	-1	12.4
-1	-1	-1	1	-1	-1	-1	-1	-6.6

-1	-1	-1	-1	1	-1	-1	-1	-8.2
-1	-1	-1	-1	-1	1	-1	-1	-7.6
-1	-1	-1	-1	-1	-1	1	-1	2.4
-1	-1	-1	-1	-1	-1	-1	1	8.8

]

```
%Define Inputs and Outputs

x = data(: , 1:8);
y = data(: , 9);

%IDentif.AI Analysis

result = stepwiselm(x , y, ‘quadratic’, 'ResponseVar', 'Inhibition', 'PredictorVars', {‘Y-27632’, ‘LY-294002’,
‘H-1152’, ‘SB203580’, ‘Irosustat’, ‘TM5441’, ‘Losartan’, ‘SD-208’});
```

Data S1.

This file contains a spreadsheet with three tabs showing the predicted percent αSMA reduction of all 2-drug, 3-drug, and 4-drug combinations in male and female VICs.

REFERENCES AND NOTES

1. E. Aikawa, J. D. Hutcheson, The developmental origin of calcific aortic stenosis, *N. Engl. J. Med.* **386**, 1372–1374 (2022).

2. N. Bhatia, S. S. Basra, A. H. Skolnick, N. K. Wenger, Aortic valve disease in the older adult. *J. Geriatr. Cardiol.* **13**, 941–944 (2016).

3. R. L. J. Osnabrugge, D. Mylotte, S. J. Head, N. M. Van Mieghem, V. T. Nkomo, C. M. LeReun, A. J. J. C. Bogers, N. Piazza, A. P. Kappetein, Aortic stenosis in the elderly: Disease prevalence and number of candidates for transcatheter aortic valve replacement: A meta-analysis and modeling study. *J. A. Coll. Cardiol.* **62**, 1002–1012 (2013).

4. S. R. Kapadia, M. B. Leon, R. R. Makkar, E. M. Tuzcu, L. G. Svensson, S. Kodali, J. G. Webb, M. J. Mack, P. S. Douglas, V. H. Thourani, V. C. Babaliaros, H. C. Herrmann, W. Y. Szeto, A. D. Pichard, M. R. Williams, G. P. Fontana, D. C. Miller, W. N. Anderson, J. J. Akin, M. J. Davidson, C. R. Smith, PARTNER trial investigators, 5-year outcomes of transcatheter aortic valve replacement compared with standard treatment for patients with inoperable aortic stenosis (PARTNER 1): A randomised controlled trial. *Lancet* **385**, 2485–2491 (2015).

5. M. A. Daubert, N. J. Weissman, R. T. Hahn, P. Pibarot, R. Parvataneni, M. J. Mack, L. G. Svensson, D. Gopal, S. Kapadia, R. J. Siegel, S. K. Kodali, W. Y. Szeto, R. Makkar, M. B. Leon, P. S. Douglas, Long-term valve performance of TAVR and SAVR: A report from the PARTNER I Trial. *JACC Cardiovasc. Imaging* **10**, 15–25 (2017).

6. M. Gozdek, G. M. Raffa, P. Suwalski, M. Kołodziejczak, L. Anisimowicz, J. Kubica, E. P. Navarese, M. Kowalewski, SIRIO-TAVI group, Comparative performance of transcatheter aortic valve-in-valve implantation versus conventional surgical redo aortic valve replacement in patients with degenerated aortic valve bioprostheses: Systematic review and meta-analysis. *Eur. J. Cardiothorac. Surg.* **53**, 495–504 (2018).

7. P. J. Goleski, M. Reisman, C. W. Don, Reversible thrombotic aortic valve restenosis after valve-in-valve transcatheter aortic valve replacement. *Catheter. Cardiovasc. Interv.* **91**, 165–168 (2018).

8. A. Kumar, K. Sato, J. Narayanswami, K. Banerjee, K. Andress, C. Lokhande, D. Mohananey, A. K. Anumandla, A. R. Khan, A. C. Sawant, V. Menon, A. Krishnaswamy, E. M. Tuzcu, W. A. Jaber, S. Mick, L. G. Svensson, S. R. Kapadia, Current society of thoracic surgeons model reclassifies mortality risk in patients undergoing transcatheter aortic valve replacement. *Circ. Cardiovasc. Interv.* **11**, e006664 (2018).

9. O. Aksoy, A. Cam, S. S. Goel, P. L. Houghtaling, S. Williams, E. Ruiz-Rodriguez, V. Menon, S. R. Kapadia, E. M. Tuzcu, E. H. Blackstone, B. P. Griffin, Do bisphosphonates slow the progression of aortic stenosis? *J. Am. Coll. Cardiol.* **59**, 1452–1459 (2012).

10. K. L. Chan, K. Teo, J. G. Dumesnil, A. Ni, J. Tam, ASTRONOMER Investigators, Effect of lipid lowering with rosuvastatin on progression of aortic stenosis: Results of the aortic stenosis progression observation: Measuring effects of rosuvastatin (ASTRONOMER) trial. *Circulation* **121**, 306–314 (2010).

11. S. J. Cowell, D. E. Newby, R. J. Prescott, P. Bloomfield, J. Reid, D. B. Northridge, N. A. Boon, Scottish Aortic Stenosis and Lipid Lowering Trial, Impact on Regression (SALTIRE) Investigators, A randomized trial of intensive lipid-lowering therapy in calcific aortic stenosis. *N. Engl. J. Med.* **352**, 2389–2397 (2005).

12. A. B. Rossebø, T. R. Pedersen, K. Boman, P. Brudi, J. B. Chambers, K. Egstrup, E. Gerdts, C. Gohlke-Bärwolf, I. Holme, Y. A. Kesäniemi, W. Malbecq, C. A. Nienaber, S. Ray, T. Skjærpe, K. Wachtell, R. Willenheimer, SEAS Investigators, Intensive lipid lowering with simvastatin and ezetimibe in aortic stenosis. *N. Engl. J. Med.* **359**, 1343–1356 (2008).

13. M. E. Arnegard, L. A. Whitten, C. Hunter, J. A. Clayton, Sex as a biological variable: A 5-year progress report and call to action. *J. Womens Health* **29**, 858–864 (2020).

14. S. Vallabhajosyula, S. P. Ponamgi, S. Shrivastava, P. R. Sundaragiri, V. M. Miller, Reporting of sex as a variable in cardiovascular studies using cultured cells: A systematic review. *FASEB J.* **34**, 8778–8786 (2020).

15. Y. Sritharen, M. Enriquez-Sarano, H. V. Schaff, G. Casaclang-Verzosa, J. D. Miller, Pathophysiology of aortic valve stenosis: Is it both fibrocalcific and sex specific? *Physiology* **32**, 182–196 (2017).

16. M. Toyofuku, T. Taniguchi, T. Morimoto, K. Yamaji, Y. Furukawa, K. Takahashi, T. Tamura, H. Shiomi, K. Ando, N. Kanamori, K. Murata, T. Kitai, Y. Kawase, C. Izumi, M. Miyake, H. Mitsuoka, M. Kato, Y. Hirano, S. Matsuda, T. Inada, T. Murakami, Y. Takeuchi, K. Yamane, M. Ishii, E. Minamino-Muta, T. Kato, M. Inoko, T. Ikeda, A. Komasa, K. Ishii, K. Hotta, N. Higashitani, Y. Kato, Y. Inuzuka, C. Maeda, T. Jinnai, Y. Morikami, N. Saito, K. Minatoya, T. Kimura, CURRENT AS Registry Investigators, Sex differences in severe aortic stenosis—Clinical presentation and mortality. *Circ. J.* **81**, 1213–1221 (2017).

17. M. Voisine, M. Hervault, M. Shen, A.-J. Boilard, B. Filion, M. Rosa, Y. Bossé, P. Mathieu, N. Côté, M.-A. Clavel, Age, sex, and valve phenotype differences in fibro-calcific remodeling of calcified aortic valve. *J. Am. Heart Assoc.* **9**, e015610 (2020).

18. J. J. Thaden, V. T. Nkomo, R. M. Suri, J. J. Maleszewski, D. J. Soderberg, M.-A. Clavel, S. V. Pislaru, J. F. Malouf, T. A. Foley, J. K. Oh, J. D. Miller, W. D. Edwards, M. Enriquez-Sarano, Sex-related differences in calcific aortic stenosis: Correlating clinical and echocardiographic characteristics and computed tomography aortic valve calcium score to excised aortic valve weight. *Eur. Heart. J.* **37**, 693–699 (2016).

19. M.-A. Clavel, D. Messika-Zeitoun, P. Pibarot, S. R. Aggarwal, J. Malouf, P. A. Araoz, H. I. Michelena, C. Cueff, E. Larose, R. Capoulade, A. Vahanian, M. Enriquez-Sarano, The complex nature of discordant severe calcified aortic valve disease grading. *J. Am. Coll. Cardiol.* **62**, 2329–2338 (2013).

20. M. A. Raddatz, H. M. Gonzales, E. Farber-Eger, Q. S. Wells, B. R. Lindman, W. D. Merryman, Characterisation of aortic stenosis severity: A retrospective analysis of echocardiography reports in a clinical laboratory. *Open Heart* **7**, e001331 (2020).

21. G. K. Singh, V. Delgado, Multimodality imaging to explore sex differences in aortic stenosis. *Eur. Cardiol.* **17**, e26 (2022).

22. B. A. Aguado, C. J. Walker, J. C. Grim, M. E. Schroeder, D. Batan, B. J. Vogt, A. G. Rodriguez, J. A. Schwisow, K. S. Moulton, R. M. Weiss, D. D. Heistad, L. A. Leinwand, K. S. Anseth, Genes that escape X chromosome inactivation modulate sex differences in valve myofibroblasts. *Circulation* **145**, 513–530 (2022).

23. R. M. Gorashi, T. Baddour, S. J. Chittle, N. E. Félix Vélez, M. A. Wenning, K. S. Anseth, L. Mestroni, B. Peña, P. Guo, B. A. Aguado, Y chromosome–linked UTY modulates sex differences in valvular fibroblast methylation in response to nanoscale extracellular matrix cues. *Sci. Adv.* **11**, eads5717 (2025)

24. A. C. Liu, V. R. Joag, A. I. Gotlieb, The emerging role of valve interstitial cell phenotypes in regulating heart valve pathobiology. *Am. J. Pathol.* **171**, 1407–1418 (2007).

25. B. J. Vogt, K. S. Anseth, B. A. Aguado, Inflammatory serum factors from aortic valve stenosis patients modulate sex differences in valvular myofibroblast activation and osteoblast-like differentiation. *Biomater. Sci.* **10**, 6627 (2022).

26. H. Wang, M. W. Tibbitt, S. J. Langer, L. A. Leinwand, K. S. Anseth, Hydrogels preserve native phenotypes of valvular fibroblasts through an elasticity-regulated PI3K/AKT pathway. *Proc. Natl. Acad. Sci. U.S.A.* **110**, 19336–19341 (2013).

27. A. Astashkina, B. Mann, D. W. Grainger, A critical evaluation of in vitro cell culture models for high-throughput drug screening and toxicity. *Pharmacol. Ther.* **134**, 82–106 (2012).

28. J. Lötsch, G. Geisslinger, Low-dose drug combinations along molecular pathways could maximize therapeutic effectiveness while minimizing collateral adverse effects. *Drug Discov. Today* **16**, 1001–1006 (2011).

29. J. D. Rogers, B. A. Aguado, K. M. Watts, K. S. Anseth, W. J. Richardson, Network modeling predicts personalized gene expression and drug responses in valve myofibroblasts cultured with patient sera. *Proc. Natl. Acad. Sci. U.S.A.* **119**, e2117323119 (2022).

30. A. R. Nelson, S. L. Christiansen, K. M. Naegle, J. J. Saucerman, Logic-based mechanistic machine learning on high-content images reveals how drugs differentially regulate cardiac fibroblasts. *Proc. Natl. Acad. Sci. U.S.A.* **121**, e2303513121 (2024).

31. A. T. L. Truong, A. Blasiak, M. Egermark, D. Ho, "AI for Drug Repurposing in the Pandemic Response" in *Artificial Intelligence in Covid-19*, N. Lidströmer, Y. C. Eldar, Eds. (Springer International Publishing, 2022) pp. 59–84.

32. E. Tekin, C. White, T. M. Kang, N. Singh, M. Cruz-Loya, R. Damoiseaux, V. M. Savage, P. J. Yeh, Prevalence and patterns of higher-order drug interactions in Escherichia coli. *npj Syst. Biol. Appl.* **4**, 31 (2018).

33. A. Torkamannia, Y. Omidi, R. Ferdousi, SYNDEEP: A deep learning approach for the prediction of cancer drugs synergy. *Sci. Rep.* **13**, 6184 (2023).

34. F. Rafiei, H. Zeraati, K. Abbasi, P. Razzaghi, J. B. Ghasemi, M. Parsaeian, A. Masoudi-Nejad, CFSSynergy: Combining feature-based and similarity-based methods for drug synergy prediction. *J. Chem. Inf. Model.* **64**, 2577–2585 (2024).

35. D. J. J. Poon, L. M. Tay, D. Ho, M. L. K. Chua, E. K.-H. Chow, E. L. L. Yeo, Improving the therapeutic ratio of radiotherapy against radioresistant cancers: Leveraging on novel artificial intelligence-based approaches for drug combination discovery. *Cancer Lett.* **511**, 56–67 (2021).

36. J. J. Lim, J. Goh, M. B. M. A. Rashid, E. K. Chow, Maximizing efficiency of artificial intelligence-driven drug combination optimization through minimal resolution experimental design. *Adv. Therap.* **3**, 1900122 (2020).

37. I. Al-Shyoukh, F. Yu, J. Feng, K. Yan, S. Dubinett, C.-M. Ho, J. S. Shamma, R. Sun, Systematic quantitative characterization of cellular responses induced by multiple signals. *BMC Syst. Biol.* **5**, 88 (2011)

38. L. M. Chong, P. Wang, V. V. Lee, S. Vijayakumar, H. Q. Tan, F. Q. Wang, T. D. Y. Y. Yeoh, A. T. L. Truong, L. W. J. Tan, S. B. Tan, K. Senthil Kumar, E. Hau, B. A. Vellayappan, A.

Blasiak, D. Ho, Radiation therapy with phenotypic medicine: Towards N-of-1 personalization. *Br. J. Cancer* **131**, 1–10 (2024).

39. A. Blasiak, L. W. J. Tan, L. M. Chong, X. Tadeo, A. T. L. Truong, K. Senthil Kumar, Y. Sapanel, M. Poon, R. Sundar, S. De Mel, D. Ho, Personalized dose selection for the first Waldenström macroglobulinemia patient on the PRECISE CURATE.AI trial. *npj Digit. Med.* **7**, 223 (2024).

40. T. Kee, C. Weiyan, A. Blasiak, P. Wang, J. K. Chong, J. Chen, B. T. T. Yeo, D. Ho, C. L. Asplund, Harnessing CURATE.AI as a digital therapeutics platform by identifying N-of-1 learning trajectory profiles. *Adv. Therap.* **2**, 1900023 (2019).

41. A. J. Pantuck, D. Lee, T. Kee, P. Wang, S. Lakhotia, M. H. Silverman, C. Mathis, A. Drakaki, A. S. Belldegrun, C. Ho, D. Ho, Modulating BET bromodomain inhibitor ZEN-3694 and enzalutamide combination dosing in a metastatic prostate cancer patient using CURATE.AI, an artificial intelligence platform. *Adv. Therap.* **1**, 1800104 (2018).

42. T. B. Toh, D. K. H. Thng, N. Bolem, B. A. Vellayappan, B. W. Q. Tan, Y. Shen, S. Y. Soon, Y. L. E. Ang, N. Dinesh, K. Teo, V. D. W. Nga, S. W. Low, P. L. Khong, E. K.-H. Chow, D. Ho, T. T. Yeo, A. L. A. Wong, Evaluation of ex vivo drug combination optimization platform in recurrent high grade astrocytic glioma: An interventional, non-randomized, open-label trial protocol. *PLOS ONE* **19**, e0307818 (2024).

43. J. Goh, S. De Mel, M. M. Hoppe, M. B. Mohd Abdul Rashid, X. Y. Zhang, P. Jaynes, E. Ka Yan Ng, N. D. B. Rahmat, Jayalakshmi, C. X. Liu, L. Poon, E. Chan, J. Lee, Y. L. Chee, L. P. Koh, L. K. Tan, T. G. Soh, Y. C. Yuen, H.-Y. Loi, S.-B. Ng, X. Goh, D. Eu, S. Loh, S. Ng, D. Tan, D. M. Z. Cheah, W. L. Pang, D. Huang, S. Y. Ong, C. Nagarajan, J. Y. Chan, J. C. H. Ha, L. P. Khoo, N. Somasundaram, T. Tang, C. K. Ong, W.-J. Chng, S. T. Lim, E. K. Chow, A. D. Jeyasekharan, An ex vivo platform to guide drug combination treatment in relapsed/refractory lymphoma. *Sci. Transl. Med.* **14**, eabn7824 (2022).

44. A. Blasiak, A. T. L. Truong, A. Remus, L. Hooi, S. G. K. Seah, P. Wang, D. H. Chye, A. P. C. Lim, K. T. Ng, S. T. Teo, Y.-J. Tan, D. M. Allen, L. Y. A. Chai, W. J. Chng, R. T. P. Lin, D. C.

B. Lye, J. E.-L. Wong, G.-Y. G. Tan, C. E. Z. Chan, E. K.-H. Chow, D. Ho, The IDentif.AI-x pandemic readiness platform: Rapid prioritization of optimized COVID-19 combination therapy regimens. *npj Digit. Med.* **5**, 83 (2022).

45. A. Blasiak, J. J. Lim, S. G. K. Seah, T. Kee, A. Remus, D. H. Chye, P. S. Wong, L. Hooi, A. T. L. Truong, N. Le, C. E. Z. Chan, R. Desai, X. Ding, B. J. Hanson, E. K.-H. Chow, D. Ho, IDentif.AI: Rapidly optimizing combination therapy design against severe acute respiratory syndrome coronavirus 2 (SARS-Cov-2) with digital drug development. *Bioeng. Transl. Med.* **6**, e10196 (2021).

46. D. Mukherjee, P. Wang, L. Hooi, V. Sandhu, K. You, A. Blasiak, E. K.-H. Chow, D. Ho, P. L. R. Ee, Addressing antimicrobial resistance with the IDentif.AI platform: Rapidly optimizing clinically actionable combination therapy regimens against nontuberculous mycobacteria. *Theranostics* **12**, 6848–6864 (2022).

47. M. Li, K. You, P. Wang, L. Hooi, Y. Chen, A. Siah, S. Tan, J. Teo, O. Ng, K. Marimuthu, I. Venkatachalam, A. Blasiak, E. K. Chow, D. Ho, Y. Gan, Discovery of broad-spectrum repurposed drug combinations against Carbapenem-Resistant Enterobacteriaceae (CRE) through artificial intelligence (AI)-driven platform. *Adv. Therap.* **7**, 2300332 (2024).

48. P. Wang, D. Ho, Deep learning and drug discovery for healthy aging. *ACS Cent. Sci.* **9**, 1860–1863 (2023).

49. M. B. M. A. Rashid, T. B. Toh, L. Hooi, A. Silva, Y. Zhang, P. F. Tan, A. L. Teh, N. Karnani, S. Jha, C.-M. Ho, W. J. Chng, D. Ho, E. K.-H. Chow, Optimizing drug combinations against multiple myeloma using a quadratic phenotypic optimization platform (QPOP). *Sci. Transl. Med.* **10**, eaan0941 (2018).

50. K. You, N. B. M. Yazid, L. M. Chong, L. Hooi, P. Wang, I. Zhuang, S. Chua, E. Lim, A. Z. X. Kok, K. Marimuthu, S. Vasoo, O. T. Ng, C. E. Z. Chan, E. K.-H. Chow, D. Ho, Flash optimization of drug combinations for Acinetobacter *baumannii* with IDentif.AI-AMR. *npj Antimicrob. Resist.* **3**, 12 (2025).

51. J.-H. Chen, W. L. K. Chen, K. L. Sider, C. Y. Y. Yip, C. A. Simmons, β-Catenin mediates mechanically regulated, transforming growth factor-β1–induced myofibroblast differentiation of aortic valve interstitial cells. *Arterioscler. Thromb. Vasc. Biol.* **31**, 590–597 (2011).

52. A. Blasiak, A. T. L. Truong, P. Wang, L. Hooi, D. H. Chye, S.-B. Tan, K. You, A. Remus, D. M. Allen, L. Y. A. Chai, C. E. Z. Chan, D. C. B. Lye, G.-Y. G. Tan, S. G. K. Seah, E. K.-H. Chow, D. Ho, IDentif.AI-omicron: Harnessing an AI-derived and disease-agnostic platform to pinpoint combinatorial therapies for clinically actionable anti-SARS-CoV-2 intervention. *ACS Nano* **16**, 15141–15154 (2022).

53. H. Xu, J. Jaynes, X. Ding, Combining two-level and three-level orthogonal arrays for factor screening and response surface exploration. *Stat. Sin.* DOI:10.5705/SS.2012.210 (2013).

54. A. M. Porras, N. C. A. van Engeland, E. Marchbanks, A. M. Cormack, C. V. C. Bouten, M. H. Yacoub, N. Latif, K. S. Masters, Robust generation of quiescent porcine valvular interstitial cell cultures. *J. Am. Heart Assoc.* **6**, e005041 (2017).

55. C. J. Walker, C. Crocini, D. Ramirez, A. R. Killaars, J. C. Grim, B. A. Aguado, K. Clark, M. A. Allen, R. D. Dowell, L. A. Leinwand, K. S. Anseth, Nuclear mechanosensing drives chromatin remodelling in persistently activated fibroblasts. *Nat. Biomed. Eng.* **5**, 1485–1499 (2021).

56. B. A. Aguado, J. C. Grim, A. M. Rosales, J. J. Watson-Capps, K. S. Anseth, Engineering precision biomaterials for personalized medicine. *Sci. Transl. Med.* **10**, eaam8645 (2018).

57. K. Fogg, N.-H. Tseng, S. R. Peyton, P. Holeman, S. M. Loughlin, J. P. Fisher, A. Sutton, A. Shikanov, J. S. Gnecco, K. M. Knight, E. M. Slaby, J. D. Weaver, N. N. Hashemi, Y. Zhang, M. D. House, B. J. Vogt, B. A. Aguado, J. C. Bradford, J. L. Robinson, P. K. Thomas, A. G. Lau, M. L. Oyen, Roadmap on biomaterials for women's health. *J. Phys. Mater.* **6**, 012501 (2023).

58. F. Wu, A. Gao, J. Liu, Y. Shen, P. Xu, J. Meng, T. Wen, L. Xu, H. Xu, High modulus conductive hydrogels enhance in vitro maturation and contractile function of primary cardiomyocytes for uses in drug screening. *Adv. Healthc. Mater.* **7**, e1800990 (2018).

59. J. Bruns, T. Egan, P. Mercier, S. P. Zustiak, Glioblastoma spheroid growth and chemotherapeutic responses in single and dual-stiffness hydrogels. *Acta Biomater.* **163**, 400–414 (2023).

60. T. He, S. Qiao, C. Ma, Z. Peng, Z. Wu, C. Ma, L. Han, Q. Deng, T. Zhang, Y. Zhu, G. Pan, FEK self-assembled peptide hydrogels facilitate primary hepatocytes culture and pharmacokinetics screening. *J. Biomed. Mater. Res. B. Appl. Biomater.* **110**, 2015–2027 (2022).

61. K. M. Mabry, S. Z. Payne, K. S. Anseth, Microarray analyses to quantify advantages of 2D and 3D hydrogel culture systems in maintaining the native valvular interstitial cell phenotype. *Biomaterials* **74**, 31–41 (2016).

62. A. Rutkovskiy, A. Malashicheva, G. Sullivan, M. Bogdanova, A. Kostareva, K. Stensløkken, A. Fiane, J. Vaage, Valve interstitial cells: The key to understanding the pathophysiology of heart valve calcification. *J. Am. Heart Assoc.* **6**, e006339 (2017).

63. J. Liao, X. Li, Y. Gan, S. Han, P. Rong, W. Wang, W. Li, L. Zhou, Artificial intelligence assists precision medicine in cancer treatment. *Front. Oncol.***12**, 998222 (2023).

64. K. M. Watts, W. Nichols, W. J. Richardson, Computational screen for sex-specific drug effects in a cardiac fibroblast signaling network model. *Sci. Rep.* **13**, 17068 (2023).

65. C. Cui, C. Huang, K. Liu, G. Xu, J. Yang, Y. Zhou, Y. Feng, G. Kararigas, B. Geng, Q. Cui, Large-scale in silico identification of drugs exerting sex-specific effects in the heart. *J. Transl. Med.* **16**, 236 (2018).

66. Y. J. Shimada, J. J. Passeri, A. L. Baggish, C. O'Callaghan, P. A. Lowry, G. Yannekis, S. Abbara, B. B. Ghoshhajra, R. D. Rothman, C. Y. Ho, J. L. Januzzi, C. E. Seidman, M. A. Fifer, Effects of losartan on left ventricular hypertrophy and fibrosis in patients with nonobstructive hypertrophic cardiomyopathy. *JACC Heart Fail.* **1**, 480–487 (2013).

67. R. A. Cuevas, C. C. Chu, W. J. Moorhead III, R. Wong, I. Sultan, C. S. Hilaire, Isolation of human primary valve cells for in vitro disease modeling. *J. Vis. Exp.* **170**, e62439 (2021).

68. B. A. Aguado, K. B. Schuetze, J. C. Grim, C. J. Walker, A. C. Cox, T. L. Ceccato, A.-C. Tan, C. C. Sucharov, L. A. Leinwand, M. R. G. Taylor, T. A. McKinsey, K. S. Anseth, Transcatheter aortic valve replacements alter circulating serum factors to mediate myofibroblast deactivation. *Sci. Transl. Med.* **11**, eaav3233 (2019).

69. N. E. Félix Vélez, K. Tu, P. Guo, R. R. Reeves, B. A. Aguado, Secreted cytokines from inflammatory macrophages modulate sex differences in valvular interstitial cells on hydrogel biomaterials. *J. Biomed. Mater. Res. A* **113**, e37885 (2025).

70. M. E. Schroeder, D. Batan, A. G. Rodriguez, K. F. Speckl, D. K. Peters, B. E. Kirkpatrick, G. K. Hach, C. J. Walker, J. C. Grim, B. A. Aguado, R. M. Weiss, K. S. Anseth, Osteopontin activity modulates sex-specific calcification in engineered valve tissue mimics. *Bioeng. Transl. Med.* **8**, e10358 (2023).

71. T. W. Gee, J. M. Richards, A. Mahmut, J. T. Butcher, Valve endothelial-interstitial interactions drive emergent complex calcific lesion formation in vitro. *Biomaterials* **269**, 120669 (2021).

72. A. C. Zeigler, W. J. Richardson, J. W. Holmes, J. J. Saucerman, A computational model of cardiac fibroblast signaling predicts context-dependent drivers of myofibroblast differentiation. *J. Mol. Cell. Cardiol.* **94**, 72–81 (2016).

73. H. Campo, D. Zha, P. Pattarawat, J. Colina, D. Zhang, A. Murphy, J. Yoon, A. Russo, H. B. Rogers, H. C. Lee, J. Zhang, K. Trotter, S. Wagner, A. Ingram, M. E. Pavone, S. F. Dunne, C. E. Boots, M. Urbanek, S. Xiao, J. E. Burdette, T. K. Woodruff, J. J. Kim, A new tissue-agnostic microfluidic device to model physiology and disease: The lattice platform. *Lab. Chip* **23**, 4821–4833 (2023).

74. C. Moccia, M. Cherubini, M. Fortea, A. Akinbote, P. Padmanaban, V. Beltran-Sastre, K. Haase, Mammary microvessels are sensitive to menstrual cycle sex hormones. *Adv. Sci.* **10**, e2302561 (2023).

75. A. P. Arnold, X. Chen, What does the “four core genotypes” mouse model tell us about sex differences in the brain and other tissues? *Front. Neuroendocrinol.* **30**, 1–9 (2009).

76. B. D. Fairbanks, M. P. Schwartz, A. E. Halevi, C. R. Nuttelman, C. N. Bowman, K. S. Anseth, A versatile synthetic extracellular matrix mimic via thiol- norbornene photopolymerization. *Adv. Mater.* **21**, 5005–5010 (2009).

77. K. M. Mabry, R. L. Lawrence, K. S. Anseth, Dynamic stiffening of poly(ethylene glycol)-based hydrogels to direct valvular interstitial cell phenotype in a three-dimensional environment. *Biomaterials* **49**, 47–56 (2015).

78. A. Ianevski, A. K. Giri, T. Aittokallio, SynergyFinder 3.0: An interactive analysis and consensus interpretation of multi-drug synergies across multiple samples. *Nucleic Acids Res.* **50**, W739–W743 (2022).

79. Z. P. Parra-Guillen, J. M. Cendrós Carreras, C. Peraire, R. Obach, J. Prunynosa, E. Chetaille, I. F. Trocóniz, Population pharmacokinetic modelling of irosustat in postmenopausal women with oestrogen-receptor positive breast cancer incorporating non-linear red blood cell uptake. *Pharm. Res.* **32**, 1493–1504 (2015).

80. Merck & Co. Inc. COZAAR (Losartan Potassium Tablets) 2013.
